# Leptin Receptor (LEPR) promotes proliferation, migration, and invasion and inhibits apoptosis in hepatocellular carcinoma by regulating ANXA7

**DOI:** 10.1186/s12935-020-01641-w

**Published:** 2021-01-04

**Authors:** He Huang, Jun Zhang, Fei Ling, Yuhong Huang, Min Yang, Yao Zhang, Yuanyi Wei, Qingqing Zhang, Honghai Wang, Lin Song, Ying Wu, Jiayu Yang, Jianwu Tang

**Affiliations:** 1 Department of Pathology, College of Basic Medical Sciences, Dalian Medical University, 9 W. Lushun South Road, Dalian, 116044 Liaoning China; 2 Department of Pathology, Tangshan People’s Hospital, 65 Shengli Road, Tangshan, 063001 Hebei China; 3 Helen Diller Family Comprehensive Cancer Center, University of California, San Francisco, CA 94158 USA

**Keywords:** LEPR, ANXA7, apoptosis, cell proliferation, cell migration, interacting proteins, hepatocellular carcinoma

## Abstract

**Background:**

Leptin Receptor (LEPR) has been suggested to have several roles in cancer metastasis. However, the role of LEPR and its underlying mechanisms in lymphatic metastasis of hepatocarcinoma have not yet been studied.

**Methods:**

We performed bioinformatics analysis, qRT-PCR, western blotting, immunohistochemistry, immunofluorescence, enzyme-linked immunosorbent, coimmunoprecipitation assays and a series of functional assays to investigate the roles of LEPR in hepatocellular carcinoma.

**Results:**

We discovered that LEPR was highly expressed in liver cancer tissues, and the expression of LEPR in Hca-F cells was higher than that in Hca-P cells. Furthermore, LEPR promotes the proliferation, migration and invasion and inhibits the apoptosis of hepatocarcinoma lymphatic metastatic cells. Further studies indicated that LEPR interacts with ANXA7. Mechanistically, LEPR regulated ERK1/2 and JAK2/STAT3 expression via ANXA7 regulation.

**Conclusions:**

These findings unveiled a previously unappreciated role of LEPR in the regulation of lymphatic metastatic hepatocellular carcinoma, assigning ANXA7-LEPR as a promising therapeutic target for liver cancer treatments.

## Background

Hepatocellular carcinoma (HCC) is one of the most common gastrointestinal cancers and possesses high heterogeneity and dynamic progression [1, 2]. Lymphatic metastasis, as the first step of the metastatic process, is an important determinant of the prognosis of hepatocellular carcinoma [3]. However, in-depth research exploring specific and sensitive biomarkers, lymphatic metastasis-related proteins and the molecular mechanism of lymphatic metastasis of hepatocellular carcinoma has yet to be done.

The Annexin family plays important roles in cell membrane phospholipids, membrane receptor regulation, cytoskeleton activity, membrane transport and cell adhesion [4]. Annexin A7 (ANXA7) is an important member of the Annexin family. The ANXA7 gene encodes a membrane-associated GTPase and a protein kinase C (PKC) substrate. Studies have shown that ANXA7 has Ca^2+^-dependent membrane fusion activity and can promote membrane fusion, adhesion and transport [5]. Recent studies indicate that ANXA7 is abnormally expressed in a variety of tumours. The levels of ANXA7 expression in liver cancer, breast cancer nasopharyngeal cancer, gastric cancer, colorectal cancer, and cervical squamous cell carcinomas are increasing [4–9]. In hepatocellular carcinoma, ANXA7 can promote the proliferation and migration of HCC through the MAPK/ERK signalling pathway [10]. Moreover, ANXA7 interacts with various proteins, such as ALG-2, SODD, Bcl-2, galectin-3, and RACK1, which together with ANXA7 regulate cell proliferation and metastasis [8, 11–15].

The protein LEPR, a member of the class 1 cytokine receptor family, has been suggested to play important roles in the pathogenesis of many malignant tumours, such as breast, colon, and prostate cancer. Six different isoforms of LEPR (LEPRa-f) were found [16–18]. LEPRb-mediated signalling promotes tumour growth and metastasis via downstream signalling pathways, such as the activation of PI3K, ERK1/2, and JAk2/STAT3 [19–21]. Recent studies indicate that LEPR is highly abundant in many cancers, including oesophageal, breast, gastric, colon and gastric cancer [22–24]. Accumulated evidence has indicated the role of LEPR in promoting several processes that are relevant to cancer progression, including cell proliferation, metastasis, angiogenesis and drug resistance, but its underlying mechanisms in lymphatic metastasis of hepatocarcinoma have not been studied thus far [25–28]. In this study, we explored whether LEPR promotes proliferation, migration, and invasion and inhibits apoptosis in hepatocellular carcinoma by regulating ANXA7. These findings reveal new perspectives for understanding the molecular mechanism of tumour development.

## Materials and methods

### Cell culture and cell transfection

The mouse hepatocarcinoma cell lines Hca-F and Hca-P were established and maintained by our laboratory in our laboratory [3, 7, 8]. The cells were cultured in RPMI 1640 medium supplemented with 10% foetal bovine serum (Gibco, USA) at 37 °C with 5% CO_2_. The cells were divided into six groups: shRNA-LEPR plasmids were transfected into Hca-F cells (F_LEPR−DOWN_ cells); plasmids containing a sequence unrelated to LEPR were transfected into Hca-F cells (F_LEPR−NC_ cells); ANXA7 plasmids were transfected into Hca-P cells (P_ANXA7−UP_ cells); plasmids containing a sequence unrelated to ANXA7 were transfected into Hca-F cells (F_ANXA7−NC_ cells); plasmids containing a sequence unrelated to ANXA7 were transfected into Hca-P cells (P_ANXA7−NC_ cells), and shRNA-ANXA7 plasmids were transfected into Hca-F cells (F_ANXA7−DOWN_ cells). The cells in the different groups were added to a 6-well plate one hour prior to transfection, transfected with 1 µg DNA and 2 µl Lipofectamine 2000 per well (Invitrogen, USA), and cultured for 48 h in RPMI-1640 medium according to the manufacturer’s directions. Transfection efficiency was detected by fluorescence microscopy at 48 h. The expression of ANXA7 and LEPR mRNA was assessed by qRT-PCR, while protein analysis was performed by western blotting.

### qRT-PCR

According to the manufacturer’s instructions, total RNA was extracted from cells by TRIzol (Invitrogen, USA) and measured with a Nanodrop 2000 spectrophotometer (Thermo Scientific, USA). cDNA was synthesized using 1 mg of RNA and a PrimeScript™ RT reagent Kit with gDNA Eraser (TaKaRa, Japan). mRNA expression was measured by qRT-PCR (MX3005P, USA) using SYBR Premix Ex Taq II (TaKaRa, Japan). The LEPR primers were 5’-CGAGTGGTCGGCACCTTCT-3′ (forward) and 5′-TCCTGCGTTGCCTTGGGT-3′ (reverse). The ANXA7 primers were 5′-AGGTCGGTGTGAACGGATTTG-3′ (forward) and 5′-TGTAGACCATGTAGTTGAGGTCA-3′ (reverse). The GAPDH primers were 5′-GGACCTGACCTGCCGTCTAG-3′ (forward) and 5′-GTAGCCCAGGATGCCCTTGA-3′ (reverse). The relative mRNA expression was determined using the comparative 2 −ΔΔCt method.

### Western blot (WB) analysis

The eight groups of cells, namely, F_ANXA7−DOWN_, P_ANXA7−UP_, F_ANXA7−NC_, P_ANXA7−NC_, Hca-F, Hca-P, F_LEPR−DOWN_, and F_LEPR−NC_ cells, were collected. Equal amounts of protein from each group were separated for ANXA7 and LEPR expression analysis using 12% sodium dodecyl sulfate-polyacrylamide gel electrophoresis (SDS-PAGE) and were transferred to polyvinylidene fluoride (PVDF) membranes (Millipore, USA). The membranes were incubated with polyclonal antibodies against ANXA7 (Abcam, USA, 1:1000), LEPR (Proteintech, China, 1:500) and GAPDH (Proteintech, China, 1:500) overnight at 4 °C followed by secondary antibodies (IRDye 800CW donkey anti-mouse/rabbit; LI-COR, USA 1:12,000) for 1 h at room temperature. Images were obtained using an Odyssey Imaging System (LI-COR Biosciences, USA) and analysed by ImageJ software.

### Enzyme-linked immunosorbent assay

Supernatants from the cells (F_ANXA7−DOWN_, P_ANXA7−UP_, F_ANXA7−NC_, P_ANXA7−NC_, Hca-F and Hca-P) were harvested and stored at -80 °C for measurement. The concentration of mouse LEPR was analysed by enzyme-linked immunosorbent assay (ELISA) using a commercial kit (Elabscience, USA) following the manufacturer’s instructions.

### Immunofluorescence assay

Cells (F_ANXA7−DOWN_, P_ANXA7−UP_, F_ANXA7−NC_, P_ANXA7−NC_, Hca-F and Hca-P) spread on poly-L-lysine-coated slides were fixed in 4% paraformaldehyde for 15 min. The cells were then blocked by incubation with goat serum (ZSGB-BIO, China) for one hour after incubation with rabbit anti-LEPR (Proteintech, USA, 1:200) and mouse anti-Annexin A7 (Abcam, USA, 1:200) overnight at 4 °C. The secondary antibody (DyLight 594 AffiniPure Donkey Anti-Rabbit/Mouse; Abbkine, USA, 1:50) was used at a 1:100 dilution for one hour at 37 °C. The cell nuclei were stained with DAPI (Beyotime, China) for 5 min at a concentration of 5 µg/ml and examined under a fluorescence microscope (Olympus, Japan).

### Coimmunoprecipitation

Coimmunoprecipitation (co-IP) was performed according to the standard procedures of an Immunoprecipitation Kit KIP-1 (IP Kit, Proteintech Group). Briefly, the cells were lysed with lysis buffer containing protease inhibitors, and the cell lysates were incubated overnight at 4 °C with primary antibody to generate immune complexes. The targeted immune complexes were captured using Protein A/G agarose, and then the elutes were submitted to immunoblotting.

### Cell proliferation assays

The cells (Hca-F, F_LEPR−DOWN_ and F_LEPR−NC_) were collected and inoculated into 96-well plates at a density of 1 × 10^4^ cells/ml in each group, and 10 µl of CCK8 solution (Dojindo Laboratories, Kumamoto, Japan) was added into each well at the time points of 0 hour (h), 24 h, 48 h, 72 h and 96 h. The numbers of cells in six replicate wells were measured at 450 nm by Multiskan (Thermo USA).

### Cell migration and invasion assays

A total of 2.5 × 10^5^ cells/well (Hca-F, F_LEPR−DOWN_ and F_LEPR−NC_) were seeded without serum into the upper chambers of insert Transwell chambers (8 µm pore size, Corning, USA), and medium supplemented with 30% serum was added into the lower chamber. After 24 hours of culture, the cells that migrated into the lower side were stained with 0.1% crystal violet and assessed using light microscopy. The invasion assay was observed with transwell chambers precoated with Matrigel (BD Bioscience, San Jose, CA, USA) to produce an artificial basement membrane. The membranes were rehydrated with 60 µl of FBS-free medium. Further steps were performed as described in the migration assay above.

### Flow cytometry assay

Apoptosis was detected by a FITC Annexin V Apoptosis Detection Kit (Dojindo Laboratories, Kumamoto, Japan). The cells (Hca-F, F_LEPR−DOWN_ and F_LEPR−NC_) were harvested and resuspended in 500 µl of binding buffer. The cells were stained with 5 µl of FITC-Annexin-V and 5 µl of propidium iodide for 30 min in the dark. Apoptotic cells were analysed by an Accuri C6 Flow Cytometer (BD Biosciences, Franklin Lakes, NJ, USA).

### Statistical analysis

Each group of experiments was repeated 3 times. The experimental data were statistically analysed using SPSS 17.0 software. A t-test was used to compare between groups. The measurement data were expressed as the mean ± standard deviation, and the comparison of means was analysed by one-way analysis of variance; the significance level was α = 0.05.

## Results

### LEPR was highly expressed in liver cancer tissues and Hca-F cells

**Fig. 1:**
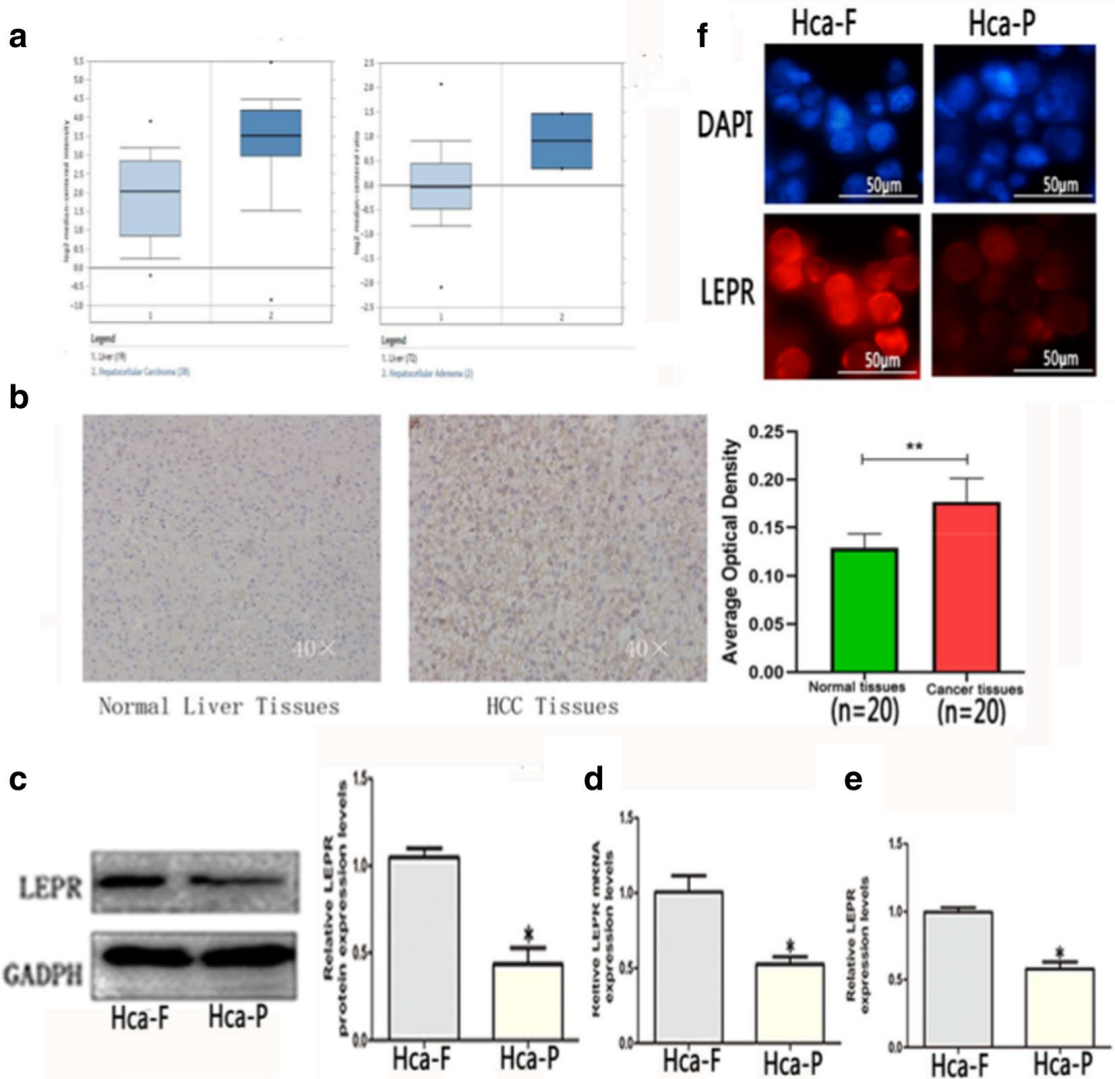
LEPR was highly expressed in liver cancer tissues and Hca-F cells. The Oncomine database (**a**) analysis of the expression of LEPR in liver cancer tissues compared with normal liver tissue. IHC staining image (**b**) analysis of the expression of LEPR in normal tissues and liver cancer tissues. qRT-PCR (**c**), WB (**d**), immunofluorescence (**e**) and enzyme-linked immunosorbent (**f**) analysis of the expression of LEPR in Hca-F and Hca-P cells

In order to investigate the role of LEPR in tumorigenesis, we examined the liver cancer database Oncomine to evaluate the differential expression of LEPR [21]. The Oncomine database analysis indicated that cancer tissues had a significantly higher expression level of LEPR than normal samples (Fig. 1a). Similar results were also found via immunohistochemistry analysis (Fig. 1b). The LEPR levels in Hca-F cells were 1.90-fold and 2.44-folder higher at the mRNA (Fig. 1c) and protein levels (Fig. 1d), respectively, than those in Hca-P cells. Similarly, cytoimmunofluorescence indicated that Hca- F cells also exhibited much higher LEPR expression than Hca-P cells (Fig. 1e). In addition, in contrast to Hca-P cells, ELISA demonstrated that LEPR secretion in the cell supernatant was unregulated in Hca-F cells (Fig. 1f), and the LEPR levels in Hca-F cell supernatant were approximately 1.72-fold higher than those in Hca-P cell supernatant. Together, these results confirmed that that LEPR expression is enriched in liver cancer.

### LEPR affects the biological behaviour of lymphatic metastatic hepatocellular carcinoma cells

**Fig. 2:**
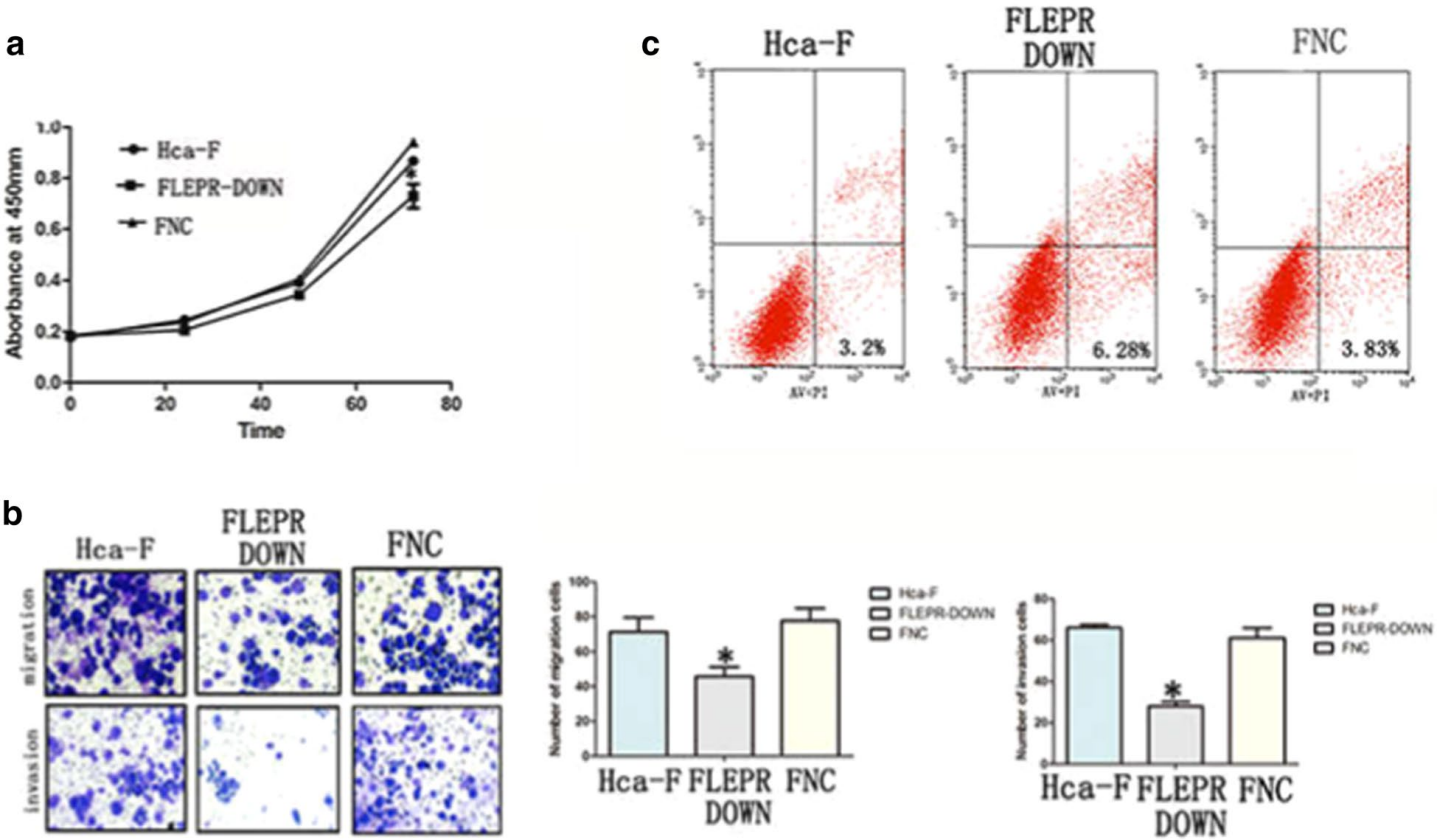
Knockdown of LEPR inhibited the proliferation, migration, and invasion and promoted the apoptosis of Hca-F cells. CCK-8 (**a**) analysis of cell proliferation potential in Hca-F cells; transwell migration and invasion assays (**b**) to analyse cell migration and invasion potentials in Hca-F cells; flow cytometry (**c**) analysis of apoptotic cells in Hca-F cells

To assess the contribution of LEPR in lymphatic metastatic hepatocarcinoma cells, cell proliferation assays, transwell migration and invasion assays, and flow cytometry assays were conducted. Cell proliferation assays showed significant inhibition of cell proliferation in the F_LEPR−DOWN_ group compared to the two control groups, Hca-F cells and F_NC_ cells (Fig. 2a). The number of cells in the F_LEPR−DOWN_ group was 75% that of the F_NC_ group at 48 h, and the number of cells in the F_LEPR−DOWN_ group was 77% that of the F_NC_ group at 72 h. The number of F_LEPR−DOWN_ cells that passed through the filter (46 ± 9) was lower than that of Hca-F cells (71 ± 14) and F_NC_ cells (78 ± 13; *p* < 0.05; Fig. 2b). Hca-F and F_NC_ cells showed similar migration abilities. The migration ability of F_LEPR−DOWN_ cells was decreased by 35% compared to that of Hca-F cells. The number of F_LEPR−DOWN_ cells that passed through the filter (28 ± 4) was lower than that of Hca-F cells (61 ± 9) and F_NC_ cells (66 ± 3; *p* < 0.05; Fig. 2b). Hca-F and F_NC_ cells showed similar invasion abilities. The invasion ability of F_LEPR−DOWN_ cells was decreased by 54% compared to that of Hca-F cells.

To further confirm the role of LEPR in hepatocarcinoma cells, we detected the percentage of apoptotic cells using flow cytometry with Annexin V/PI double staining. In the F_LEPR−DOWN_ group, the Annexin-V-positive and PI-negative portions representing the early apoptotic pattern were significantly increased to 6.28% compared with 3.2% in the Hca-F group and 3.83% in the F_NC_ group (Fig. 2c). Together, these results indicated that LEPR promoted the proliferation, invasion and migration and inhibited the apoptosis of hepatocarcinoma cells.

### LEPR interact with ANXA7

**Fig. 3:**
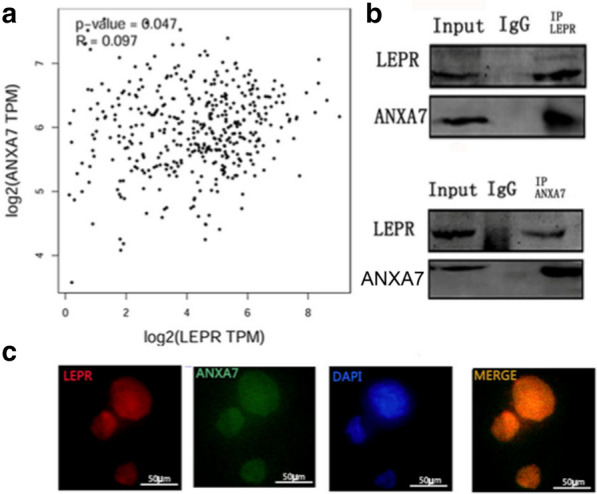
LEPR interacted with ANXA7. The GEPIA database (**a**) demonstrated that LEPR and ANXA7 interacted with each other; coimmunoprecipitation (**b**) analysis showed that LEPR formed a complex with ANXA7 in Hca-F cells; immunofluorescence staining assays (**c**) analysis showed that LEPR and ANXA7 were colocalized

GEPIA database analysis demonstrated that LEPR and ANXA7 interacted with each other (Fig. 3a). Similar results were also found via coimmunoprecipitation and immunofluorescence staining assays in Hca-F cells. LEPR were found to be coimmunoprecipitated with ANXA7 (Fig. 3b). Furthermore, immunofluorescence staining assays revealed that both proteins colocalized (Fig. 3c). Collectively, these results showed that LEPR could interact with ANXA7.

### LEPR regulated ERK1/2 and JAK2/STAT3 expression via ANXA7 regulation

**Fig. 4:**
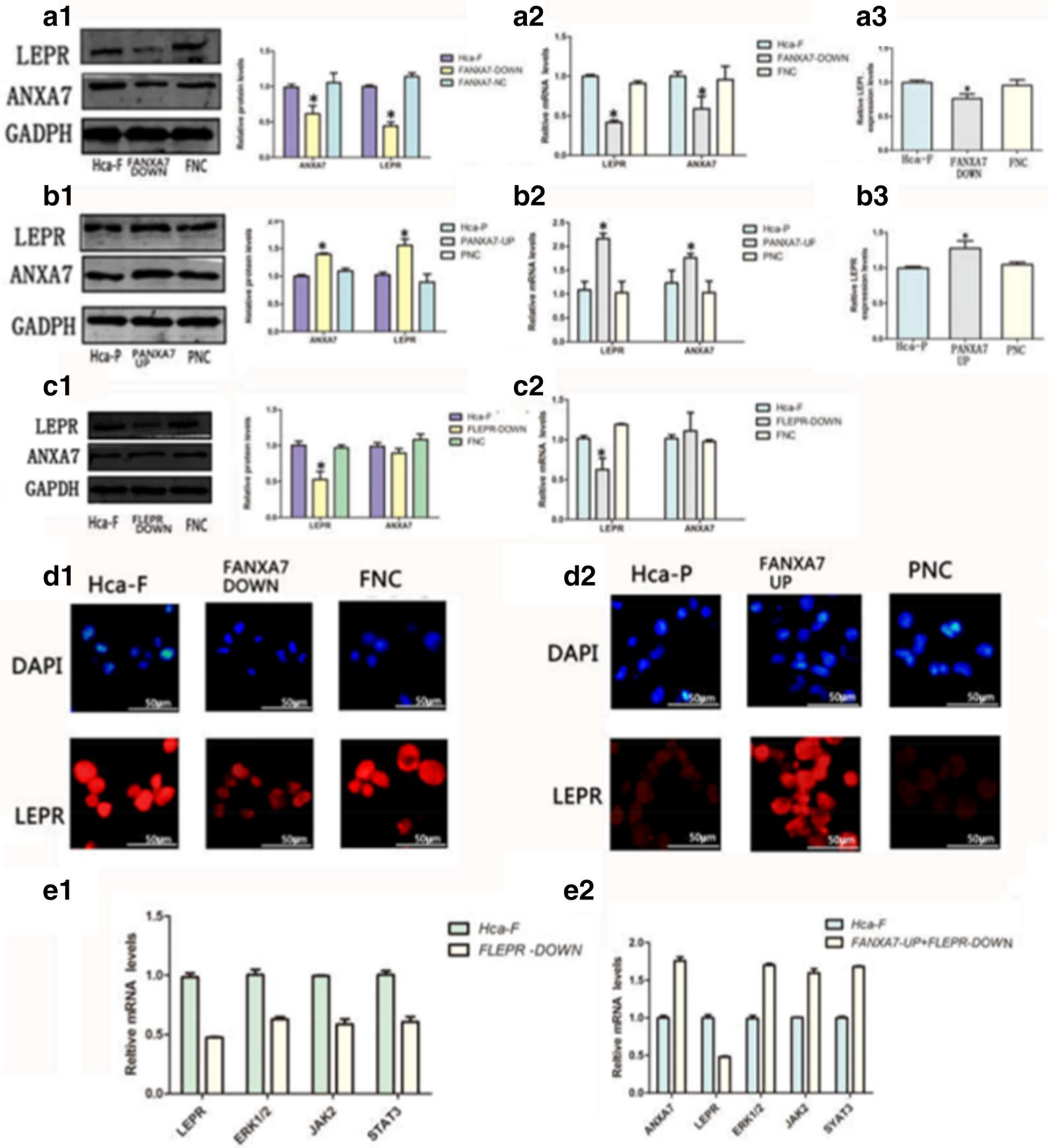
LEPR regulated ERK1/2 and JAK2/STAT3 expression via ANXA7 regulation in hepatocarcinoma cells. qRT-PCR (**a2**), WB (**a1**), enzyme-linked immunosorbent (**a3**) and immunofluorescence (**d1**) analysis of LEPR expression, in Hca-F, FANXA7-DOWN, FNC cells; qRT-PCR (**b2**) and WB (**b1**), enzyme-linked immunosorbent (**b3**) and immunofluorescence (**d2**) analysis of LEPR expression in Hca-P, PANXA7-UP, PNC cells; qRT-PCR (**c2**) and WB (**c1**) analysis of ANXA7 expression in Hca-F, FLEPR-DOWN, FNC cells; qRT-PCR (**e1**) analysis of the ERK1/2, JAK2, STAT3 expression level in Hca-F, FLEPR-DOWN cells; qRT-PCR (**e2**) analysis of the ERK1/2, JAK2, STAT3 expression level in Hca-F, FANXA7-UP + LEPR-DOWN cells

We found that there was a significant positive relationship between the expression of LEPR and ANXA7 gene regulation. First, ANXA7 knockdown reduced the expression of LEPR, whereas ANXA7 upregulation promoted the expression of LEPR. After ANXA7 knockdown, the ANXA7 level of F_LEPR−DOWN_ cells was decreased by 42% (mRNA) and 38.5% (protein) compared to that of F_NC_ cells (P < 0.05). The LEPR level of F_LEPR−DOWN_ cells was decreased by 59% (mRNA) and 55.5% (protein) compared to that of F_NC_ cells (P < 0.05) (Fig. 4a1, a2). After the upregulation of ANXA7, the ANXA7 level in P_ANXA7−UP_ cells was 1.4-fold (mRNA and protein) higher than that in P_NC_ cells (P < 0.05). The LEPR level in P_ANXA7−UP_ cells was 2.6-fold (mRNA) and 1.55-fold (protein) higher than that in P_NC_ cells (P < 0.05) (Fig. 4b1, b2). Furthermore, immunofluorescence assays showed similar results (Fig. 4d1, d2). ELISA demonstrated that ANXA7 upregulation elevated LEPR secretion in the cell supernatant (Fig. 4a3, b3). To further confirm the relationship between LEPR and ANXA7, we performed qRT-PCR and western blot analysis for Hca-F cells with LEPR knocked down, which showed that the ANXA7 expression level did not significantly change. These findings suggested that LEPR did not influence the expression of ANXA7 (P > 0.05) (Fig. 4c1, c2). To further explore the mechanism by which LEPR expression affected lymphatic metastasis of hepatocarcinoma, we found that LEPR knockdown reduced the expression of ERK1/2, JAK2 and STAT3, whereas ANXA7 upregulation partly restored the expression level of ERK, JAK2, and STAT3 in Hca-F cells. After LEPR knockdown, the LEPR level of F_LEPR−DOWN_ cells was decreased by 48% compared to that of Hca-F cells (P < 0.05). The ERK level of F_LEPR−DOWN_ cells was decreased by 63% compared to that of Hca-F cells (P < 0.05). The JAK2 level of F_LEPR−DOWN_ cells was decreased by 58.5% compared to that of Hca-F cells (P < 0.05). The STAT3 level of F_LEPR−DOWN_ cells was decreased by 60.5% compared to that of Hca-F cells (P < 0.05) (Fig. 4e1). After upregulation of ANXA7 and knockdown of LEPR, the ANXA7 level in F_ANXA7−UP+LEPR−DOWN_ cells was 1.79-fold higher than that in F cells (P < 0.05). The LEPR level of F_ANXA7−UP+LEPR−DOWN_ cells was decreased by 49% compared to that of Hca-F cells (P < 0.05). The ERK level in F_ANXA7−UP+LEPR−DOWN_ cells was 1.70-fold higher than that in Hca-F cells (P < 0.05). The JAK2 level in F_ANXA7−UP+LEPR−DOWN_ cells was 1.595-fold higher than that in Hca-F cells (P < 0.05). The STAT3 level in F_ANXA7−UP+LEPR−DOWN_ cells was 1.68-fold higher than that in Hca-F cells (P < 0.05) (Fig. 4e2). Taken together, these results indicated that LEPR regulated ERK1/2 and JAK2/STAT3 expression via ANXA7 regulation.

## Discussion

Recent studies have suggested that obesity is associated with an increased risk of several cancer types, including kidney, breast, liver, colon, gastric, gallbladder, oesophagus and pancreatic cancer. Leptin has been extensively identified as a potential molecule involved in obesity-related cancer [29, 30]. Cancer cells release leptin and express leptin receptor (LEPR), which suggests that leptin/LEPR signalling plays roles in tumour progression. The protein LEPR is expressed in many tissues, including adipocytes, thalamus cells, thyroid follicular epithelial cells, gastric epithelial cells, adrenal cortical cells, and organs, including the heart, lung, liver, kidney, and prostate [31]. Moreover, LEPR is expressed at higher levels in many tumour tissues than in normal tissues, including oesophageal cancer, colon cancer, breast cancer and gastric cancer cells [22–24]. In this study, we demonstrated the overexpression of LEPR in liver cancer tissue. Additionally, similar results were found in the Oncomine database. However, the underlying mechanisms of LEPR in lymphatic metastasis of hepatocarcinoma remain unclear.

The Hca-F (lymph node metastasis > 75%) and Hca-P (lymph node metastasis < 25%) cell lines are subclones derived from the same parent cells of mouse hepatocarcinoma ascitic cells by our laboratory many years ago. Therefore, sharing the same genetic background, the two cell lines are ideal models for revealing potential biomarkers related to lymphatic metastasis [3, 7, 11–15]. Previously, our laboratory used a gene chip technique to identify differentially expressed genes, and LEPR was more highly expressed in Hca-F cells than in Hca-P cells, which indicates that they are candidate genes for mouse hepatocarcinoma lymphatic metastasis [32]. In this study, we further confirmed that LEPR expression levels were increased in Hca-F cells compared to Hca-P cells. Simultaneously, the concentration of LEPR secreted in the cell supernatant had the same trend as the expression of LEPR within hepatocarcinoma cells. The above results suggest that LEPR may be involved in lymphatic metastasis.

LEPR is a single transmembrane protein belonging to the superfamily of cytokine receptors distributed in many tissues [33, 34]. In recent years, it was verified that LEPR is associated with carcinogenesis. In human cell lines and animals, LEPR was reported to be associated with increased tumour cell proliferation, metastasis, angiogenesis and drug resistance [35]. Clinically, enhanced expression of LEPR was observed in human oesophageal, breast, gastric, colon and gastric cancer tissues and could predict cancer progression in bladder, endometrial and ovarian cancer [36–38]. In this study, we found that LEPR may be involved in lymphatic metastasis. Later, we conducted cell proliferation assays, transwell migration and invasion assays, and flow cytometry assays to assess the contribution of LEPR to lymphatic metastatic hepatocarcinoma cells. Initially, CCK-8 assay showed that cell proliferation ability markedly decreased following the depletion of LEPR in Hca-F cells. Furthermore, transwell migration and invasion assays revealed that the knockdown of LEPR expression in Hca-F cells obviously inhibited migration and invasion abilities. Flow cytometry assays showed that LEPR knockdown enhanced cell apoptosis. Collectively, these results indicate that LEPR promotes the proliferation, migration and invasion and inhibits the apoptosis of hepatocarcinoma lymphatic metastatic cells.

Membrane-linked protein A7 (ANXA7) is associated with tumours, which are known to be lymphatic metastasis-related proteins [10]. ANXA7 is associated with the cell membrane transport, signal transduction, proliferation and invasion of tumour cells [5]. ANXA7 does not consistently function in different types of cancer. ANXA7 might specifically function as a tumour promoter candidate in liver cancer, breast cancer, nasopharyngeal carcinoma, gastric cancer, and colorectal cancer. ANXA7 might act as a tumour suppressor gene in prostate, melanoma and glioblastoma cancer [39–45]. In our laboratory, we found that the suppression of ANXA7 in Hca-F cells decreased proliferation, migration and invasion and increased the number of apoptotic cells. Many proteins have been reported to interact with ANXA7, such as ALG-2, SODD, Bcl-2, Galectin-3, and RACK1, which together with ANXA7 regulate cell proliferation and metastasis [8, 11–15]. In our laboratory, we used immunoprecipitation combined with mass spectrometry to identify proteins that interact with ANXA7 in mouse hepatoma cells, including LEPR (unpublished data). In this study, we further identified the interaction of LEPR with ANXA7.

To further explore the mechanism by which LEPR expression affected lymphatic metastasis of hepatocarcinoma, cells with ANXA7 overexpression or ANXA7 knocked down were used to study the expression of LEPR. Experiments showed that ANXA7 knockdown reduced both the mRNA and protein levels of LEPR, whereas ANXA7 upregulation increased the expression of LEPR. However, the expression of ANXA7 did not significantly change after LEPR was knocked down. Previous studies have demonstrated that LEPR also promotes cell proliferation, migration and invasion by modulating intracellular signalling pathways, such as the ERK1/2, JAk2/STAT3 and PI3K pathways. In human hepatocarcinoma cells, researchers have found that leptin/LEPR signalling triggers the JAK2-PI3K/Akt-MEK/ERK1/2 pathway, which results in the upregulation of cyclinD1 expression and downregulation of Bax expression that accelerates cell cycle progression to stimulate cell proliferation and prevents cells from undergoing the apoptotic G1-S transition [33]. Leptin and its receptor LEPR promote the proliferation and metastasis of gallbladder carcinoma, which may participate in the regulation of MMPs and the VEGF family through the SOCS3/JAK2/STAT3 pathways [46]. In this study, LEPR knockdown reduced the expression of ERK1/2, JAK2 and STAT3, whereas ANXA7 upregulation partly restored the expression levels of ERK1/2, JAK2, and STAT3 in Hca-F cells. Collectively, these results indicate that LEPR regulated ERK1/2 and JAK2/STAT3 expression via ANXA7 regulation.

## Conclusions

This represents the first study reporting that LEPR promoted proliferation, migration, and invasion and inhibited apoptosis in hepatocellular carcinoma by regulating ANXA7. This finding shows the potential of LEPR as a novel therapeutic target for hepatocellular carcinoma, while the LEPR-ANXA7 complex may serve as a potential target for tumour growth and metastasis prevention, which influences the occurrence and development of liver cancer.

## Data Availability

The datasets used and/or analyzed in this study are available from the corresponding author upon reasonable request.

## Abbreviations

HCC: Hepatocellular carcinoma
ANXA7: AnnexinA7
LEPR: Leptin Receptor receptor
F_LEPR−DOWN_: Plasmids of shRNA-LEPR transfected into Hca-F cells
F_LEPR−NC_cells: Plasmids of LEPR unrelated sequence transfected into Hca-F cells
P_ANXA7−UP_: Plasmids of ANXA7 transfected into Hca-P cells
F_ANXA7−NC_cells: Plasmids of ANXA7 unrelated sequence transfected into Hca-F cells
P_ANXA7−NC_ cells: Plasmids of ANXA7 unrelated sequence transfected into Hca-P cells
F_ANXA7−DOWN_: Plasmids of shRNA-ANXA7 transfected into Hca-F cells
